# Growing in red: impact of different light spectra and lighting conditions on lentil microgreens growth in vertical farming

**DOI:** 10.3389/fpls.2024.1515457

**Published:** 2024-12-23

**Authors:** Marlus Dias Silva, Jaqueline Martins Vasconcelos, Fábia Barbosa da Silva, Adriano Soares de Oliveira Bailão, Ítalo Moraes Rocha Guedes, Márcio da Silva Vilela, Adriano Carvalho Costa, Márcio Rosa, Fabiano Guimarães Silva

**Affiliations:** ^1^ Laboratory of Advanced Studies in Vertical Agriculture, Goiano Federal Institute of Education, Science and Technology, Rio Verde, Brazil; ^2^ Laboratory of Computational Intelligence, Goiano Federal Institute of Education, Science and Technology, Rio Verde, Brazil; ^3^ Brazilian Agricultural Research Corporation (EMBRAPA), Embrapa Hortaliças, Brasília, Brazil; ^4^ Laboratory of Automation, Simulation and Control, Goiano Federal Institute of Education, Science and Technology, Rio Verde, Brazil; ^5^ Graduate Program in Plant Production, University of Rio Verde, Rio Verde, Brazil

**Keywords:** vertical farming, lighting regimes, *Lens culinaris*, microgreens, constant light, gaussian curve, photosynthetic efficiency, artificial lighting

## Abstract

Vertical Farming Systems (VFS) emerge as an approach to optimize plant growth in urban and controlled environments, by enabling sustainable and intensive production in reduced spaces. VFS allow for greater control over growing conditions, such as light, temperature and humidity, resulting in higher quality crops and with less use of resources, such as water and fertilizers. This research investigates the effects of different lighting regimes (Constant and Gaussian) and spectral qualities (white, RBW, blue and red) on the growth, photosynthesis, and biomass accumulation of lentil microgreens (*Lens culinaris*) in VFS. The results demonstrate that constant lighting regimes, particularly under red, white, and RBW lights, significantly increase biomass production and energy efficiency. On the other hand, the Gaussian regime promotes the accumulation of bioactive compounds such as carotenoids, especially under red light. Chlorophyll content and the photochemical coefficient (qP) also varied across treatments, with significant variations between lighting regimes and spectral combinations. Tailored lighting strategies, adjusted to specific production goals, have the potential to enhance both productivity and nutritional quality in VFS. The analysis contained in the research provides relevant information for optimizing lighting management in controlled agricultural environments, providing practical applications to improve harvest performance.

## 1 Introduction

Vertical farming, also known as the Vertical Farming System (VFS), has gained prominence as an innovative approach to plant cultivation in urban and controlled environments, optimizing space usage and enabling efficient food production in areas with land limitations Bantis and Koukounaras (2024). The precise control of factors such as lighting, temperature, and humidity enhances plant growth, improving not only productivity but also the nutritional and biochemical quality of the cultivated plants. VFS are particularly advantageous in regions where natural resources are limited, promoting a more sustainable and efficient form of agriculture van Delden et al. (2021).

One of the main obstacles in vertical farming is managing artificial lighting conditions, as light plays a fundamental role in the process of photosynthesis and plant development. The use of LEDs (Light Emitting Diodes) has proven to be a good alternative for cultivation, allowing fine-tuning of the intensity, duration, and spectrum of light, factors that directly influence photosynthesis and biomass accumulation Yudina et al. (2023). Light spectra such as red and blue, for instance, have been widely recognized for their beneficial effects on plant growth. Monochromatic red light can stimulate cell elongation, while blue light promotes compactness and the production of photosynthetic pigments. However, the combination of different spectra, such as RBW (red, blue, and white), has also shown good results, regulating photosynthesis to enhance plant growth and quality Budavári et al. (2024); Ciriello et al. (2023); Zhang et al. (2022).

The selection of light spectrum is essential for optimizing photosynthetic efficiency, growth, and biomass accumulation in controlled cultivation systems. Research has shown that variations in UV-A LED wavelengths and exposure durations can significantly enhance bioactive compounds production in mustard microgreens without compromising growth Brazaitytė et al. (2019). Furthermore, a balanced combination of red, green, and blue light has been found to optimize growth and metabolite accumulation in radish microgreens, while UV-A and far-red light improve antioxidant properties in a cultivar-specific manner Garegnani et al. (2024). Light intensity also plays a critical role in agronomic traits and phytochemical composition: lower intensities promote chlorophyll content and cotyledon expansion, whereas higher intensities enhance antioxidant activity and total phenolic content, influencing microgreen quality and productivity Flores et al. (2024).

Thus, it can be seen that the choice of light spectrum is essential for optimizing photosynthetic efficiency, growth, and biomass accumulation in controlled cultivation systems. Therefore, the use of differentiated light spectra, tailored to the needs of each crop and stage of development, is crucial for optimizing production in vertical farming systems, ensuring healthy growth and higher quality agricultural products Nájera et al. (2022); Boucher et al. (2023).

Microgreens, in particular, stand out in the context of VFS due to their short growth cycle, ranging from 5 to 10 days from germination to harvest Bantis and Koukounaras (2024). Due to their high added value, these vegetables are becoming increasingly common in plant factories with artificial lighting (PFAL) in urban areas Kozai and Niu (2020); Orsini et al. (2020); Boonmee et al. (2024). Furthermore, the high sowing density and low height of these plants make them ideal for cultivation in small spaces, allowing for continuous production throughout the year, regardless of the seasons Cowden et al. (2024).

In this context, lentils (*Lens culinaris*), a widely cultivated legume valued for its high nutritional content and ease of cultivation, are an advantageous crop for studying the effects of different light regimes and qualities on biomass accumulation, photosynthetic efficiency, and pigment production Preiti et al. (2024). Studies have shown that lentils are highly responsive to changes in environmental conditions, including variations in light quality, which directly influence their carbohydrate metabolism and overall yield. These characteristics make them an ideal candidate for optimization in vertical cultivation systems Bhandari et al. (2016). Additionally, lentil microgreens have been highlighted for their nutritional value and adaptability to controlled environments Priti et al. (2021), for example, observed higher levels of flavonoids, carotenoids, and ascorbic acid in lentil microgreens grown under semi-controlled conditions. These findings align with the growing interest in microgreens due to their distinct flavor profiles and high nutrient content Dubey et al. (2024). Furthermore, lentils’ ability to regulate antioxidant and photoprotective metabolism under stress conditions reinforces their suitability for controlled environments Saini et al. (2024).

Although several studies have assessed the impact of different light spectra on the quality of microgreens, most focus exclusively on constant lighting regimes—i.e., on how light is provided over time. Furthermore, the works are limited to investigating variations in photoperiods. However, could it be possible that different lighting regimes also influence the growth of microgreens in diverse ways? Constant regimes, which provide a uniform light intensity, and modulated regimes, such as Gaussian curves that simulate natural light variations throughout the day, could affect the development of these vegetables? These adjustments could promote the optimization of photosynthesis and biomass accumulation? It is hypothesized that the lighting regimes constant light and modulation with Gaussian curves combined with variations in light spectrum, may distinctly influence the growth, photosynthesis, and biomass production of lentils cultivated in vertical farming systems. Thus, this research seeks to explore the impact of lighting regimes (constant and Gaussian), in association with spectral variations (white light, RBW, blue, and red), on the growth and quality of lentils cultivated in vertical farming environments. A detailed understanding of these lighting effects may provide important information for optimizing cultivation practices in these systems, ensuring greater productivity and quality of harvests.

## 2 Materials and methods

### 2.1 Experimental setup and cultivation conditions

**Figure 1:**
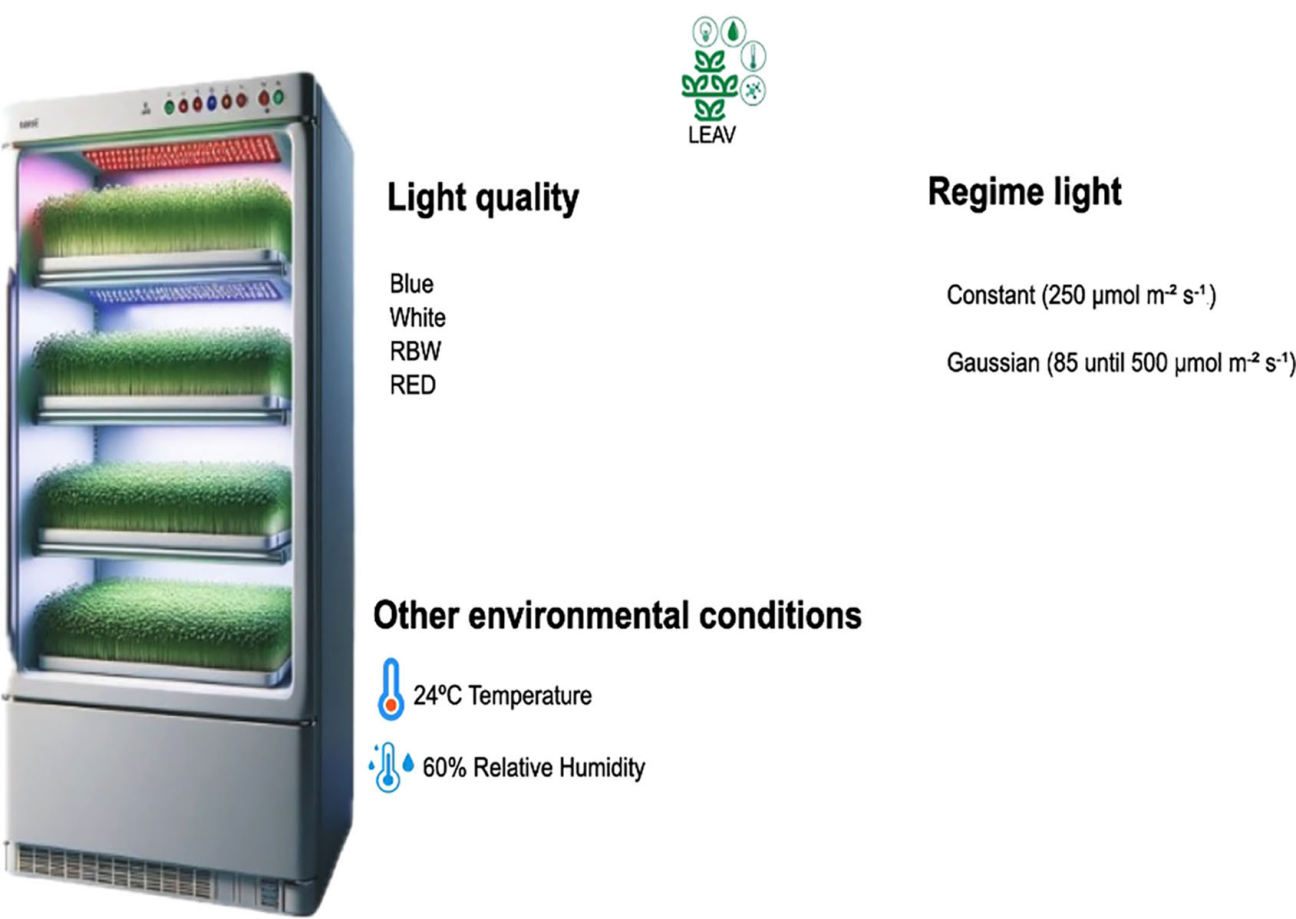
Representation of the cultivation environment.

The experiment was conducted at the Laboratory of Advanced Studies in Vertical Agriculture, Goiano Federal Institute of Education, Science and Technology, Rio Verde, Brazil. Thirteen grams (13 g) of lentil seeds (*Lens culinaris*) Yoki^®^ (Brazil, BR), approximately 200 seeds for each technical replicate, were distributed in plastic containers measuring 20 cm in length, 14 cm in width, and 6.5 cm in height, containing 100 g of the commercial substrate *Bioplant Plus^®^*, previously moistened with 25 mL of water and kept in the dark for 12 hours. Subsequently, the containers were placed in a growth chamber (Spectral Int^®^, Rio Verde, Brazil) (**Figure 1**) that is 1.80 meters tall and 65 cm wide, equipped with four shelves containing luminaires measuring 45×55cm, with 55 LEDs evenly distributed and installed at a distance of 22 cm between shelves. The luminaires were programmed to provide a distinct spectral composition of light: cool white 6500 K (400-700 nm), red (600-700 nm, peak at 660 nm), blue (400-500 nm, peak at 440 nm), and RBW (Red: Blue : White - spectral composition: 70.5% red, 8.5% green, and 21.0% blue).

Lentil microgreens were cultivated under a photoperiod of 12 hours (06:30 to 18:30) in two lighting regimes: constant and Gaussian curve (**Supplementary Figure S11**). In the constant regime, the light intensity was maintained at 250 *µ*mol m^−2^ s^−1^, while in the Gaussian regime, using an automatic dimming system, the intensity varied from 85 to 500 *µ*mol m^−2^ s^−1^. In both regimes, a supply of 10.80 mol m^−2^ day^−1^ of DLI (*Daily Light Integral*) was ensured. Light intensity and spectral composition were measured using a LI-180 light spectrometer (LI-COR, Nebraska, USA). Cultivation in the constant regime took place from March 8 to 18, 2024, followed by the Gaussian regime from March 21 to April 1, 2024, with a duration of 10 days each.

Irrigation was adjusted throughout the experiment according to the growth of the seedlings and the water needs of the substrate. Each experimental unit corresponded to a replicate, represented by a plastic tray containing approximately 200 lentil seeds treated with a specific combination of lighting regime and spectral quality. In the first 7 days, 25 mL of water was applied daily to each experimental unit. From the eighth day onwards, the amount of water was increased to 35 mL per day due to the greater development of the seedlings and the consequent reduction in substrate moisture. The average air temperature in the growth chamber was maintained at 24°C ± 0.5, with relative humidity controlled at 60% ± 0.5 during the cultivation period (**Supplementary Figures S9**, **S10**).

The design was completely randomized in a 2x4 factorial scheme with two lighting regimes (Constant and Gaussian) and four spectral qualities of light (white, blue, red, and RBW), with six repetitions, totaling 48 experimental units. After 10 days of sowing, fluorescence assessment of chlorophyll *a*, photosynthetic pigments, growth, and biomass was conducted.

### 2.2 Growth and biomass assessment of lentils

The planning of the analysis to evaluate plant growth consists of multiple replicates. Each replicate includes 15 plants selected for measuring hypocotyl length (CHL), number of leaves (3), and average leaf area. The measurements of these variables were obtained from photographs of the plants, analyzed using the ImageJ software, as highlighted by Schneider et al. (2012).

The remaining plants from each replicate were used to determine the fresh and dry mass of the hypocotyl and leaves. Samples were weighed on an analytical balance to obtain the fresh mass and then dried in an oven (Tecnal, TE-394/1, Brazil) at a temperature of 65°C for 48 hours to determine the dry mass.

### 2.3 Chlorophyll *a* fluorescence

The chlorophyll *a*fluorescence parameters were obtained using the IMAGING PAM modulated fluorometer (MAXI version) and the Imaging Win software (Heinz Walz GmbH, Effeltrich, Germany). The seedlings were individually fixed in a holder at a distance of 18.5 cm from a charge-coupled device (CCD) camera connected to a fluorescence device. Measurements were taken from the adaxial portion of the leaves, adapted to the dark for 30 minutes, so that the reaction centers were fully open. Under this condition, the leaf tissues were exposed to low-intensity light (0.03 µmol m^-2^ s^-1^) to determine the initial fluorescence (Fo). Then, a saturating light pulse (> 6000 µmol m^-2^ s^-1^) was applied for 0.8 s to determine the maximum fluorescence (Fm), from which the maximum quantum yield of photosystem II was calculated (*Fv*/*Fm* = (Fm - Fo)/Fm) Genty et al. (1989).

After illuminating the sample for 40 seconds, light-adapted fluorescence was determined to measure the light-acclimated variables such as the effective quantum yield of PSII Y(II), and the non-photochemical quenching (NPQ) Kramer et al. (2004). The electron transport rate (ETR) was calculated as ETR = Y(II) × PAR × Aleaf × 0.5 Bilger et al. (1995), where PAR is the photon flux density; Aleaf is the fraction of the incident light absorbed by the leaves; and 0.5 is the fraction of excitation energy presumed to be equally distributed between PSII and PSI Laisk and Loreto (1996). Data were obtained from processing images of the median region of the leaves.

### 2.4 Photosynthetic pigments

The concentrations of the pigments were determined after extraction with dimethyl sulfoxide (DMSO) saturated with calcium carbonate (CaCO_3_) de Castro et al. (2019). The concentrations of chlorophyll *a* (Chl*a*), chlorophyll *b* (Chl*b*), and carotenoids (Cart) were determined using a UV-VIS spectrophotometer (Evolution 60S, Thermo Fisher Scientific Inc., MA, USA) at wavelengths of 665, 649, and 480 nm, respectively. The calculations were performed using the equations proposed by Wellburn (1994), and the results were expressed in *µ*g g^−1^ of fresh leaf weight.

### 2.5 Calculation of energy consumption per gram of dry matter

In the experiment, the seedlings were placed at a distance of 22 cm from the luminaires, covering an illuminated area of 0.247 m^2^. Based on the electric power of the luminaires and the illuminated area, energy consumption per square meter was calculated. For each light spectrum, the total power of the luminaires was measured as follows: 0.167653 kWh/m^2^ for white light, 0.099832 kWh/m^2^ for RBW light, 0.18536 kWh/m^2^ for blue light, and 0.075324 kWh/m^2^ for red light. This value was then divided by the estimated dry matter mass per square meter, resulting in the energy consumption per gram of dry matter. The total dry matter mass per square meter was obtained by multiplying the average dry matter mass per seedling by the total number of seedlings per square meter (1,428 seedlings), a density close to that recommended by recent studies, which indicate three seeds per square centimeter as ideal for lentil microgreens Dubey et al. (2024).

The formula for calculating energy consumption per gram of dry matter is given by:


$$E_{g}=\frac{P_{tot}\cdot A}{M_{sec}\cdot N_{m}}$$

where $-E_{g}$ is the energy consumption per gram of dry matter (kWh/g), $-P_{tot}$ is the total power of the luminaires (kWh/m^2^), - A is the illuminated area (m^2^), $-M_{sec}$ is the average dry matter mass per seedling (g), $-N_{m}$ is the number of seedlings per square meter.

### 2.6 Statistical analysis

The data analysis was carried out using the R computer program. Initially, exploratory analysis was performed considering the effects of light supply regimes, spectral light composition, and the interaction between these factors, with residual analysis conducted and outliers removed when necessary. The normality of the residuals was checked using the Shapiro-Wilk test, the homogeneity of variances was tested using the Levene test (using the car package), and the correlation of the residuals was analyzed using the Durbin-Watson test (via the lmtest package).

A two-way analysis of variance (ANOVA) was conducted to evaluate the effects of lighting regimes and spectral compositions, as well as their interaction, on the measured variables. Subsequently, analysis of variance (ANOVA) was conducted, and when effects were significant, the Tukey test at a 5% probability level was applied using the ExpDes.pt package. Aiming to expand the data analysis, Pearson linear correlation analyzes were carried out between the variables and principal components analysis (PCA). The ExpDes.pt, MVar.pt, FactoMineR, factoextra, tidyverse, corrplot, viridis, and RColorBrewer packages were utilized.

## 3 Results

**Figure 2:**
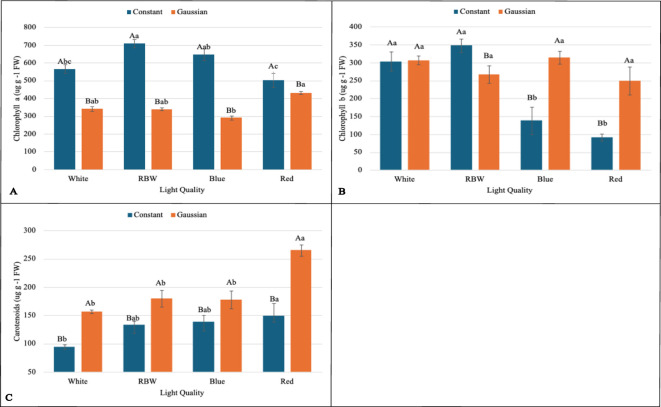
Graphs illustrating the main effects and interactions between lighting regimes (constant and Gaussian) and spectral light qualities (white, RBW, blue, and red) on pigment concentration. Values are expressed as means ± standard error, with uppercase letters indicating comparisons between lighting regimes and lowercase letters indicating comparisons between spectral qualities, according to the Tukey test (p<0,05). **(A)** Chlorophyll *a* (*µgg* ^−1^ *FW*, **(B)** Chlorophyll *b* (*µgg* ^−1^ *FW*), **(C)** Carotenoids (*µgg* ^−1^ *FW*).

**Figure 3:**
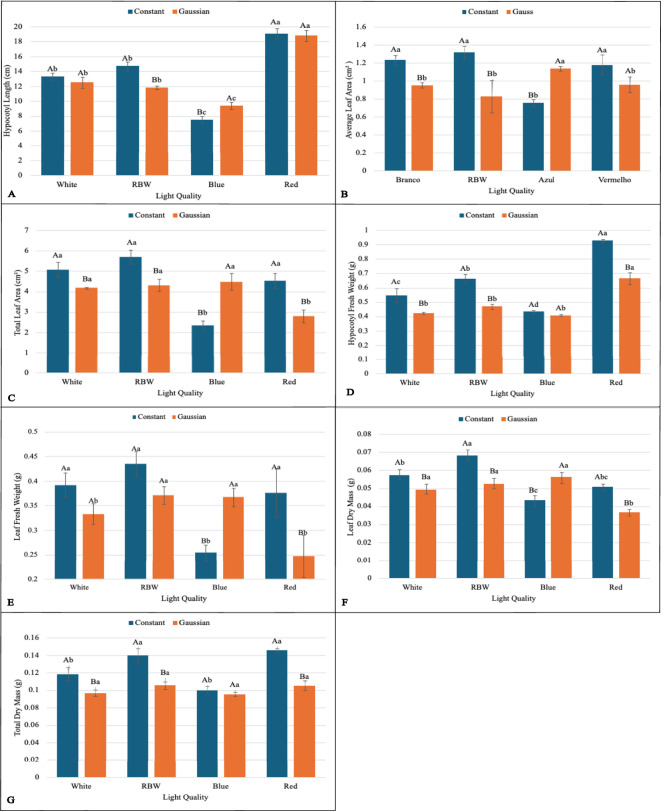
Graphs illustrating the main effects and interactions between lighting regimes (constant and Gaussian) and spectral light qualities (white, RBW, blue, and red) on growth and biomass variables. Values are presented as means ± standard error, with uppercase letters indicating comparisons between lighting regimes and lowercase letters indicating comparisons between spectral qualities, according to the Tukey test (p*<*0,05). **(A)** Hypocotyl Length (cm), **(B)** Average Leaf Area (cm^2^), **(C)** Total Leaf Area (cm^2^), **(D)** Hypocoty Fresh Weight (g), **(E)** Leaf Fresh Weight (g), **(F)** Leaf Dry Mass (g), **(G)** Total Dry Massa (g).

**Figure 4:**
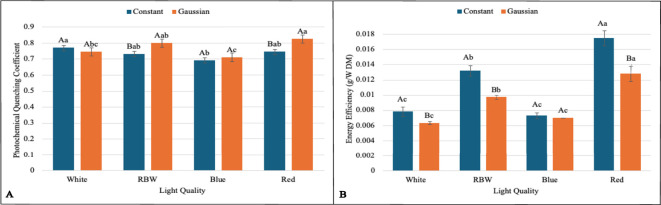
Graphs illustrating the main effects and interactions between lighting regimes (constant and Gaussian) and spectral light qualities (white, RBW, blue, and red) on photosynthetic and energy efficiency variables. Values are presented as means ± standard error, with uppercase letters indicating comparisons between lighting regimes and lowercase letters indicating comparisons between spectral qualities, according to the Tukey test (p<0,05). **(A)** Photochemical Quenching Coefficient, **(B)** Energy Efficiency (g/W DM).

The interaction between lighting regime (constant and Gaussian) and spectral qualities of light (white, RBW, blue, and red) was significant for the variables: chlorophyll *a* (Chl*a*), chlorophyll *b* (Chl*b*), carotenoid content (Cart), average leaf area (CALA), total leaf area (CTLA), hypocotyl length (CHL), hypocotyl fresh weight (HF), leaf fresh weight (LFW), leaf dry mass (LMD), total dry mass (TDM), photochemical quenching coefficient (qP), and energy efficiency (EnE) (**Figures 2**–**4**). These variables indicate how plants utilize light to convert energy into biomass and thus directly reflect photosynthetic efficiency and plant growth.

When breaking down the lighting regime in relation to the light spectrum, it was found that for chlorophyll *a* (Chl*a*), the constant regime was statistically superior to the Gaussian regime in all spectral qualities of light (white, RBW, blue, and red) (**Figure 2A**). For total leaf area (TLA), hypocotyl fresh weight (HFW), leaf dry mass (LMD), total dry mass (TDM), and energy efficiency (EnE), the constant regime was also statistically superior to the Gaussian regime under the spectra of white, RBW, and red light (**Figures 3C, D, F, G**, **4B**). For these variables, there was no statistical difference between the regimes when cultivated under blue light, except for total leaf area (TLA) and leaf dry mass (LMD), where the Gaussian regime was statistically superior to the constant regime (**Figures 3C, F**).

For average leaf area (CALA), the constant regime was superior to the Gaussian regime when seedlings were cultivated under white and RBW light. In red light, there was no significant difference between the regimes; however, when cultivated under blue light, the Gaussian regime was statistically superior to the constant regime (**Figure 3B**).

For chlorophyll *b* and hypocotyl length (CHL), the constant regime was statistically superior to the Gaussian only in the RBW light quality (**Figures 2B**, **3A**). However, the Gaussian regime had statistically higher means than the constant regime under blue and red lights for chlorophyll *b*, and there was no statistical difference between the lighting regimes in the white light spectrum. For hypocotyl length, the Gaussian regime was superior to the constant regime only under blue light (**Figures 2B**, **3A**).

For leaf fresh weight (LFW), seedlings cultivated under white and red light in the constant regime were statistically superior to the Gaussian regime. The opposite was observed in blue light cultivation, where the Gaussian regime was superior to the constant regime, and no difference between the regimes was observed under RBW light (**Figure 3E**). The carotenoid content was significantly higher in the Gaussian regime across all light types (**Figure 2C**). The photochemical quenching coefficient was also statistically superior in the Gaussian regime under RBW and red lights, with no differences between the regimes in the other light types (**Figure 4A**).

When breaking down the lighting regime within the types of light spectra, it was found that for chlorophyll *a* (Chl*a*), the constant regime was statistically superior to the Gaussian regime in all light qualities (white, RBW, blue, and red) (**Figure 2A**).

Through the breakdown of the light spectrum within each lighting regime, it was verified that there was no effect of the light spectrum in the Gaussian regime for average leaf area (CALA). In the constant regime, the white, RBW, and red colors showed higher values than blue light (**Figure 3B**). For chlorophyll *b* (Chl*b*), the different light spectra showed no statistical differences when cultivation was conducted in the Gaussian regime. In the constant regime, RBW and white did not differ from each other and were superior to blue and red (**Figure 2B**).

In the Gaussian regime for chlorophyll *a* (Chl*a*), there was a difference only between blue and red colors, with higher values for the latter (**Figure 2A**). In the constant regime, RBW showed higher values than white and red light, not differing from blue light, which also presented higher values than red light.

For carotenoids (CART) and hypocotyl fresh weight (HFW) in the Gaussian regime, the red color had a higher value than the other colors, which did not differ among themselves. In the constant regime for carotenoids, there was a difference only between red and white colors, with the latter being lower (**Figures 2C**, **3D**). In the constant regime, HFW indicated that all colors differed from each other, with the highest value for red, followed by RBW, white, and lastly blue.

In hypocotyl length (CHL) in the Gaussian regime, it was found that red light had the highest value and blue the lowest. In CHL, in both regimes, red light had the lowest value (**Figure 3A**). In energy efficiency (EnE), it was observed that in both regimes, red light had the highest value, while white and blue had lower values (**Figure 4B**).

For leaf fresh weight (LFW), in the Gaussian regime, higher values were found for RBW and blue, and lower for the other treatments. In the constant regime, blue light had a lower value than the others, which did not differ from each other (**Figure 3E**).

In total leaf area (CTLA), white, RBW, and blue lights were superior to red light in the Gaussian regime. In the constant regime, blue light showed lower values than the others, which did not differ from each other (**Figure 3C**).

For the photochemical quenching coefficient (qP) in the Gaussian regime, it was found that red light had higher values than white and blue lights. RBW light did not differ from white and was superior to blue. In the constant regime, there was a significant difference only between white and blue light, with the latter being lower (**Figure 4A**).

Total dry mass (TDM) showed higher averages under red and RBW lights compared to blue and white lights under the constant regime, which had the lowest means and were statistically equal among themselves. There was no statistical difference between the spectral light qualities in the Gaussian regime (**Figure 3G**).

When analyzing energy consumption per gram of dry matter (**Table 1**), it was observed that white light in the constant regime consumed 0.167653 $\frac{\text{kWh}}{\text{g}}$ , while in the Gaussian regime the consumption was 0.210675 $\frac{\text{kWh}}{\text{g}}$ . For RBW light, the consumption was 0.099832 $\frac{\text{kWh}}{\text{g}}$ under constant lighting and 0.135709 $\frac{\text{kWh}}{\text{g}}$ in the Gaussian regime. Blue light in the constant regime consumed 0.18536 $\frac{\text{kWh}}{\text{g}}$ , while in the Gaussian regime the consumption was 0.197157 $\frac{\text{kWh}}{\text{g}}$ . Under red light in the constant regime, the consumption was 0.075324 $\frac{\text{kWh}}{\text{g}}$ , while in the Gaussian regime it was 0.106168 $\frac{\text{kWh}}{\text{g}}$ .

**Table 1:** Energy consumption per gram of dry matter (kWh/m²) under different light spectra and lighting regimes.

Light Spectrum	Constant (kWh/m^2^)	Gaussian (kWh/m^2^)
**White**	0.167653	0.210675
**RBW**	0.099832	0.135709
**Blue**	0.185360	0.197157
**Red**	0.075324	0.106168

The bolded words highlight the light treatments used in each lighting regime presented.

Finally, for leaf dry mass (LMD), in the Gaussian regime, white, RBW, and blue lights did not differ from each other and showed higher values than red light. In the constant regime, RBW showed higher values than the other treatments, while the blue treatment had the lowest value, not differing from the red treatment (**Figure 3F**).

For the variables NPQ, Y(II), HDM, and ETR, there was no interaction effect (**Supplementary Tables S3**, **S4**), with a significant simple effect of the lighting regime and light spectrum observed for all of them. Higher values were found in the constant regime for the NPQ and HDM variables, and lower values for Y(II) and ETR compared to the Gaussian regime. For ETR, white light had lower values than the others, which did not differ from each other. For HDM, higher values were found for red light and lower for white. The Y(II) showed higher values for red and RBW and lower for blue. For NPQ, higher values were found for blue and lower for red and RBW.

In summary, we can highlight that in the experiment with the breakdown of the light regime, it was observed that the constant regime was statistically superior to the Gaussian regime in several key variables, especially under white, RBW, and red light qualities. This included chlorophyll *a* (Chl*a*), total leaf area (TLA), hypocotyl fresh weight (HFW), leaf dry mass (LMD), total dry mass (TMD), and energy efficiency (EnE). However, under blue light, there was no significant difference between the regimes, except for TLA and LMD, where the Gaussian regime surpassed the constant one. In terms of carotenoids (CART), the Gaussian regime was consistently superior regardless of light quality. Interestingly, the photochemical quenching coefficient (qP) was also higher in the Gaussian regime under RBW and red lights. For the other variables, there was variation between the regimes depending on the light quality, with the Gaussian regime performing better under blue light for some measurements.

**Figure 5:**
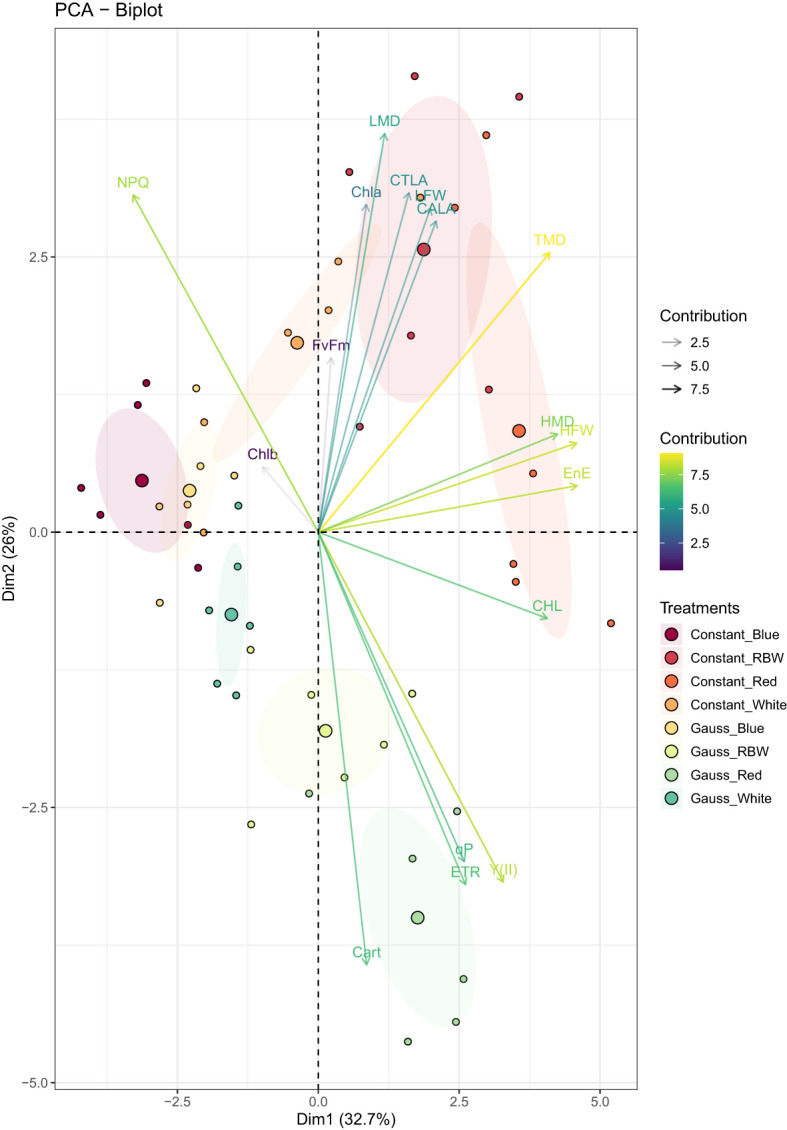
Two-dimensional scatter plot of Principal Component Analysis (PCA) representing the scores of the physiological and production variables, observations, and average values. The plot shows how different variables relate to and contribute to the variation observed in the first two principal components (PC1 and PC2). The first two principal components (PC1 and PC2) explain 32.70% and 25.95% of the total variance, respectively, accounting for a total of 58.65% of the explained variance.

From the principal component analysis, it was found that the first four components jointly explained 82.76% of the total variation in the data (**Table 2**; **Figure 5**). It was found that the first component (PC1) explained 32.70% of the variation in the data, showing a high positive correlation (—|*r*| > 0.70—) with variables such as HFW, HMD, TMD, CHL, and EnE. This indicates that these variables have the highest variability within the dataset and are directly related to each other, meaning that an increase in one causes an increase in the other. This direct relationship among the variables and their importance can be graphically observed in (**Figure 5**), where the smaller angle between the arrows indicates greater correlation, and the greater the shift in the horizontal direction, the more important the variable is within the first component.

**Table 2:** Correlation coefficients of the first four principal components (PC1, PC2, PC3, PC4) resulting from Principal Component Analysis (PCA) for different physiological and production variables.

Variables	cp1	cp2	cp3	cp4
**HFW** Hypocotyl Fresh Weight (g)	0,9	0,16	-0,34	-0,05
**LFW** Leaf Fresh Weight (g)	0,39	0,58	0,55	-0,3
**HMD** Hypocotyl Dry Mass (g)	0,84	0,18	-0,46	-0,15
**LMD** Leaf Dry Mass (g)	0,23	0,71	0,53	-0,12
**FvFm** Maximum Quantum Yield of PSII (Fv/Fm)	0,04	0,31	-0,19	0,88
**Y(II)** Effective Quantum Yield of PSII	0,65	-0,63	0,30	0,14
**NPQ** Non-Photochemical Quenching	-0,65	0,60	-0,33	-0,09
**qP** Photochemical Quenching Coefficient	0,51	-0,59	0,38	-0,42
**ETR** Electron Transport Rate)	0,51	-0,63	0,35	0,35
**Chla** Chlorophyll $a\;(\mu g\;g^{-1}FW)$	0,17	0,59	-0,42	-0,10
**Chlb** Chlorophyll $b\;(\mu g\;g^{-1}FW)$	-0,19	0,12	0,74	0,11
**Cart** Carotenoids $\left(\mu g\;g^{-1}FW\right)$	0,17	-0,77	-0,06	-0,09
**TDM** Total Dry Mass (g)	0,81	0,50	-0,09	-0,18
**EnE** Energy Efficiency (g/W DM)	0,90	0,08	-0,31	-0,01
**CTLA** Total Leaf Area ( ${\text{cm}}^{2}$ )	0,32	0,61	0,53	0,24
**CALA** Average Leaf Area ( ${\text{cm}}^{2}$ )	0,41	0,56	0,30	0,18
**CHL** Hypocotyl Length (cm)	0,80	-0,15	-0,16	0,27
Eigenvalue	5,559	4,412	2,643	1,455
Variance (%)	32,70%	25,95%	15,55%	8,56%
Cumulative Variance (%)	32,70%	58,65%	74,20%	82,76%

The bold acronyms correspond to the physiological and production variables evaluated in the experiment, indicating their respective correlation coefficients for the four main principal components (PC1, PC2, PC3, and PC4).

The second principal component explained 25.95% of the variation in the data, showing a high positive correlation with LMD and a negative correlation with Cart, indicating that this group of variables is also relevant and has an inverse correlation. The third component explained 15.55% of the variation in the data, showing a high positive correlation with chlorophyll *b*. The fourth component (PC4) explained 8.56% of the variation in the data, correlating highly with the variable F*v*F*m*.

Through the principal component analysis, it was possible to globally understand the behavior of treatments based on the evaluated variables, verifying that in the first principal component, it was possible to discriminate the treatments, showing a greater effect of light color, with a greater contrast of blue light compared to red. Red light showed higher values for the variables associated with this component, as also verified in the univariate analysis, although some variables may not have been significant.

The second principal component (PC2) indicated that the light regime (constant versus Gaussian) has an additional impact, especially within each light color, except blue. The scores close to 0 for blue light suggest that the variation in the lighting regime (constant versus dynamic) has a limited effect in this specific color, possibly due to the way blue light is absorbed and utilized in plants. In contrast, for white, RBW, and red lights, PC2 shows a trend of higher values for negatively associated variables, such as carotenoid content (CART), and lower values for positively associated variables, such as leaf dry mass (LMD), highlighting how different light regimes affect resource distribution and use in plants.

**Figure 6:**
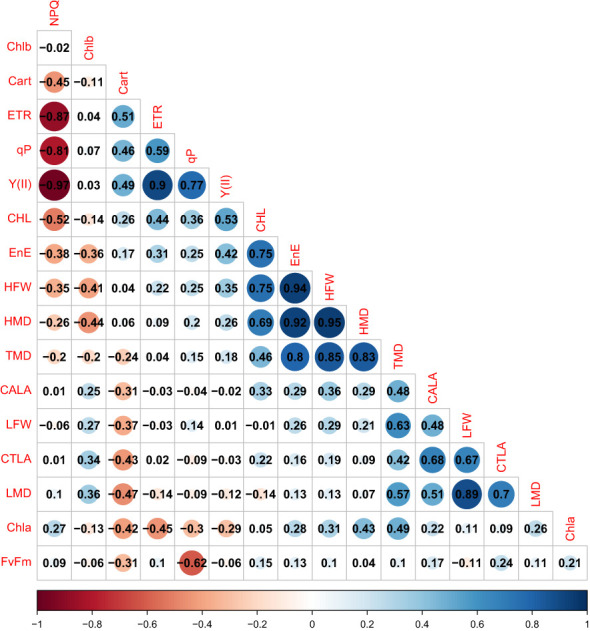
Heatmap showing the correlation coefficient (r) between the variables. Non-significant correlations are represented by white backgrounds, while negative and positive correlations are represented by shades of red and blue, respectively. The variables included are: HFW, Hypocotyl Fresh Weight; LFW, Leaf Fresh Weight; HMD, Hypocotyl Dry Mass; LMD, Leaf Dry Mass; FvFm, Maximum Quantum Yield of PSII; Y(II),Effective Quantum Yield of PSII; NPQ, Non-Photochemical Quenching; qP, Photochemical Quenching Coefficient; ETR, Electron Transport Rate); Chla, Chlorophyll *(a)*; Chlb, Chlorophyll *(b)*; Cart, Carotenoids; TDM, Total Dry Mass; EnE,Energy Efficiency; CTLA, Total Leaf Area; CALA, Average Leaf Area; and CHL, (Hypocotyl Length.

Through correlation analysis and principal component analysis (**Figure 6**), it was possible to verify the formation of groups of variables that have moderate to high positive correlations within the same group.

Group a) HFW, HMD, TMD, CHL, and EnE; b) TMD, CALA, LFW, CTLA, and LMD; c) CART, ETR, qP, and Y(II); d) NPQ. The variables in group a have moderate to low correlations with those in group b. The NPQ variable has high negative correlations with ETR, qP, and Y(II).

## 4 Discussion

Light quality and illumination regime are key environmental factors influencing in the growth, yield, and production of bioactive compounds in microgreens. However, the interactions between the light spectrum and the light regime has received less attention in the literature. Exploring these interactions offers a more comprehensive undertanding of how to optimize cultivation practices in vertical farming systems for microgreens, emphasizing their implications for growth, photosynthetic efficiency, and biomass production.

This research shows that cultivating lentil microgreens under constant red LED light (660 nm) can increase yield (**Figure 3G**), while Gaussian modulation favors carotenoid production (**Figure 2C**), a functional bioactive compound essential for human health. Similar results regarding seedling yield have been demonstrated in grapevine under constant red light Kong et al. (2024). This result may be linked to the effect of red light, which directly excites photosystem II (PSII), promoting greater efficiency in capturing and converting light into chemical energy (**Figure 4A**; **Supplementary Tables S3**, **S4**), favoring cell growth and biomass accumulation Maxwell and Johnson (2000); Yousef et al. (2021).

These responses may also be associated with the inhibitory effect of red light on the activity of the IAA oxidase enzyme, increasing endogenous auxin levels Jeong and Sivanesan (2015). Moreover, red light has a stimulating effect on endogenous gibberellins, hormones involved in mitosis and cell proliferation Toyomasu et al. (1993); Manivannan et al. (2015). These hormonal changes may contribute to increased cell elongation, resulting in greater biomass production, as shown in previous studies Poorter et al. (2019). Thus, red light, by promoting both greater photosynthetic efficiency and cell growth, proves to be an effective tool for optimizing the productive performance of microgreens Li et al. (2023).

However, the interaction between the red light spectrum and illumination regime also resulted in a significant effect, beyond just biomass production (**Figures 3A, D, G**). In the Gaussian regime, the modulation of photosynthetic responses, as observed in the PCA 5, was due to the greater influence of red light on the variables YII, qP, and ETR, and photoprotective compounds such as carotenoid concentration (**Figure 2C**). Red light can induce an increase in photoxidative response, triggering greater carotenoid production as a protective mechanism. These pigments play a critical role in photoacclimation by dissipating excess light energy and shielding cellular membranes from damage caused by reactive oxygen species (ROS) Hashimoto et al. (2016); Jahns and Holzwarth (2012); Caferri et al. (2022); Qiao et al. (2021). This adaptive response effectively optimizes light utilization under intensified light conditions (similar to the Gaussian regime), further emphasizing the role of red light in regulating photosynthetic and photoprotective pathways.

Moreover, plant pigments such as carotenoids and chlorophylls also have direct implications for the nutritional quality of plants, especially in microgreens, which are consumed at early developmental stages when the concentration of nutrients and bioactive compounds is maximized Dou et al. (2018); Pennisi et al. (2020). Carotenoids, in particular, are precursors of vitamin A and act as antioxidants, which is crucial for both photoprotection in plants and for promoting functional foods that contribute to human health Teng et al. (2024); Caferri et al. (2022). In this context, red light, which significantly increased carotenoid production, can be explored as a strategy to enhance the nutritional value of microgreens. This is achieved not only through increased biomass accumulation but also by boosting the concentration of bioactive compounds with health benefits, such as carotenoids Llorente et al. (2016).

The physiological adjustments observed under red light resulted in superior energy efficiency in both lighting regimes (constant and Gaussian), as illustrated in (**Figure 4B**). This performance is directly related to the greater ability of red light to promote photosynthesis in photosystem II (YII) (**Supplementary Tables S3**, **S4**), especially in the constant regime, where an increase in biomass production was noted. This factor is crucial for crops aiming to maximize organic matter.

The data also corroborate the energy efficiency of red light, which had the lowest energy consumption per gram of dry matter in the constant regime (0.075324 kWh/g). In contrast, the highest consumption was recorded with white light in the Gaussian regime (0.210675 kWh/g), indicating a higher energy demand related to light modulation.

These results reinforce the superiority of red light in terms of energy efficiency and biomass production, especially under the constant regime, aligning with previous studies highlighting the effectiveness of red light in optimizing photosynthesis and plant growth in controlled environments Hernández and Kubota (2016). On the other hand, the higher energy consumption of white light in the Gaussian regime may be explained by the additional energy demand to maintain light intensity variation over time, which, while promoting the synthesis of bioactive compounds, compromises efficiency in biomass accumulation Li et al. (2020).

In relation to RBW light in the constant regime, a good performance in biomass accumulation and energy efficiency is observed (**Figures 3A–F**, **4A, B**), comparable to red light. The energy consumption per gram of biomass was 0.099832 $\frac{\text{kWh}}{\text{g}}$ under constant lighting and 0.135709 $\frac{\text{kWh}}{\text{g}}$ in the Gaussian regime, trailing behind cultivation under red light and ahead of blue and white light cultivations, regardless of the cultivation type. This suggests that the mixed spectrum provided a balance between different wavelengths, optimizing both growth and the efficient use of light by plants Wang et al. (2024); Toscano et al. (2021); Nie et al. (2024). These results are also corroborated by the data presented on the photochemical efficiency of photosystem II (qP) and relative electron transport rate (ETR) (**Supplementary Tables S3**, **S4**), where both are directly linked to the plants’ ability to convert light into chemical energy, driving biomass accumulation Fork and Satoh (1986); Guenther et al. (1990); Havaux et al. (1991). These results have important applications for agriculture in controlled environments, where adjusting light regimes, including the strategic use of RBW and red light, can be an effective tool for optimizing photosynthetic efficiency and crop productivity Murchie and Niyogi (2010); Chen et al. (2018); Keller et al. (2022); Tang et al. (2024).

Some positive responses in plants under RBW may be related to the synergy with green radiation, which can promote increases in certain characteristics such as leaf area and fresh leaf mass, as phytochromes and cryptochromes, photoreceptors related to morphogenesis, are also sensitive to this radiation Folta and Maruhnich (2007); Carvalho and Folta (2016).

Blue light, on the other hand, although it demonstrated a lower effect on biomass accumulation, stood out in photosynthetic and photoprotective parameters, especially in the Gaussian regime. The adjustments promoted by blue light resulted in an increase in chlorophyll *a* (CHLA) production (**Figure 2A**) and a reduction in the effective quantum yield of PSII (Y(II)). In response to the lower YII, photoprotection and adaptation mechanisms were activated, as observed by the increase in non-photochemical quenching (NPQ) (**Supplementary Tables S3**, **S4**). This is consistent with studies showing that blue light promotes greater light absorption and efficiency in photosynthesis, but with less leaf growth, corroborating its influence on more compact morphological characteristics adapted for environments with higher light intensity Zhang et al. (2020); Ashenafi et al. (2023). This adaptive response is relevant for plants grown under conditions where light stress is a concern, such as in intensive artificial lighting systems. The ability of blue light to promote photoprotection mechanisms, along with its influence on more compact morphological development, makes it a useful option for optimizing plant resilience against environmental variations.

Despite the opposing effects observed in lentil seedlings cultivated under monochromatic red and blue light, the combination of red and blue lights, widely discussed in the literature, also emerges as an effective strategy to maximize both growth and photosynthesis in controlled systems. Previous studies have already indicated that red light stimulates stem elongation, while blue light promotes greater leaf development. Studies integrating these characteristics can result in plants with higher efficiency in light capture and, consequently, increased biomass production Fraszczak and Kula-Maximenko (2022). Our results corroborate this synergy between the two spectra, suggesting that the combined use of red and blue light can be explored to optimize both yield and quality of plants in controlled cultivation systems Ashenafi et al. (2023).

Importantly, the responses observed in this study are specific to lentil microgreens, and variations in plant species should be considered when designing light regimes. Studies with other plants, such as lettuce, radish, and spinach, have shown that different combinations of red and blue lights produce diverse effects on growth and biochemical composition Lin et al. (2013); Zukauskas et al. (2011). These interspecies variations underscore the importance of tailoring light spectra to the specific characteristics of each plant, aiming to optimize both yield and nutritional quality, particularly in large-scale cultivation systems Dou et al. (2018); Pennisi et al. (2020).

Furthermore, according to Li et al. (2011); Wei et al. (2023), during the initial seedling stage, cotyledonary reserves provide most of the energy required for growth, limiting the immediate dependence on active photosynthesis. As the seedlings mature and these reserves are depleted, the effects of different light spectra, such as the Gaussian regime, become more evident, impacting growth and photosynthetic efficiency. This has been observed in species like Arabidopsis thaliana and other plants under controlled light. These results suggest that the combination of different spectra may be more effective in later stages of development. Thus, the difference in the response observed under the Gaussian light regime, despite the use of the same DLI and spectra, can be explained by the early activation of photoprotective mechanisms, such as non-photochemical quenching (NPQ), which reduces the efficiency of converting light into chemical energy. This leads to the dissipation of energy as heat instead of being used to increase biomass production.

Despite the better photochemical performance in plants under the Gaussian regime, as evidenced by the chlorophyll a fluorescence data, this did not translate into greater leaf and total dry mass. Vialet-Chabrand et al. (2024) observed negative impacts on the accumulation of edible biomass in lettuce grown under a sinusoidal regime compared to a square wave light regime. These responses need to be further explored; however, much of this response is related to the slow process of photosynthetic induction at the beginning of the photoperiod Kaiser et al. (2014); Lawson et al. (2012).

Thus, unlike a stationary system, the gradual process of photosynthetic induction in the Gaussian or sinusoidal regime can be characterized by a temporary lag in the maximum efficiency of photosynthesis when light increases, due to the need for metabolic and biophysical adjustments in the plant, such as RuBP regeneration, Rubisco activation, and stomatal opening. Only after these processes, does photosynthesis reach an efficient steady state, where CO_2_ is assimilated optimally Vialet-Chabrand et al. (2024). According to Stamford et al. (2024), more satisfactory productivity responses in lettuce could be obtained by using the same DLI but with lower PPFD during a longer photoperiod, avoiding exposure to peaks of saturating light, where there is lower quantum efficiency.

This study highlights the importance of optimizing light conditions for lentil microgreen cultivation, emphasizing how the interaction between light regimes and spectral quality can influence both physiological performance and the production of bioactive compounds. Red light under a constant regime promoted greater biomass accumulation, aligning with previous studies emphasizing its role in optimizing photosynthesis and cell elongation in controlled systems Poorter et al. (2019); Hernández and Kubota (2016). The Gaussian regime, particularly under red and blue light, activated photoprotective mechanisms, consistent with findings linking blue light to enhanced stress responses Zhang et al. (2020). The balanced effects of RBW light, supporting growth and nutritional quality, align with literature on the synergistic potential of red and blue light combinations Ashenafi et al. (2023), while the moderate effects of white light may be related to its spectral diversity, characterized by a significantly higher proportion of green light compared to red and blue light. Although green light exhibits high leaf penetration capacity Brodersen and Vogelmann (2010), its effectiveness in activating photoreceptors such as cryptochromes and phytochromes is lower than that of blue and red light, respectively. Furthermore, green light can antagonize the activation of cryptochromes induced by blue light, reversing responses such as blue light-induced stomatal opening Smith et al. (2017), which may negatively affect gas exchange and photosynthetic capacity. In this context, white light can be advantageous in cultivation systems where uniformity and versatility are prioritized over the maximization of specific variables, such as biomass or bioactive compound production. Although the responses to Gaussian white light are subtle, it is evident that the spectral variation in this regime may have induced signaling pathways favoring the allocation of resources toward adaptation and protection mechanisms rather than growth. These findings contribute valuable insights for refining spectral combinations and illumination regimes to optimize production, particularly for high-value crops like microgreens. By advancing the understanding of spectral interactions, this study supports the development of more efficient and sustainable approaches in vertical farming, enhancing yield, nutritional quality, and valorization of these microgreens, which may also positively impact their market value Kozai et al. (2016); Pennisi et al. (2020).

## 5 Conclusions

As indicated by the obtained results, it is evident that constant and modulated lighting regimes distinctly impact the growth, photosynthesis, and biomass production in lentil microgreens. The constant regime stood out for its greater efficiency in biomass production and energy efficiency, particularly under red, RBW, and white lights. On the other hand, the Gaussian regime was more effective in inducing bioactive compounds, such as carotenoids, especially under red light, demonstrating a greater photoprotective adaptation.

The results indicate that the choice of lighting regime should be based on the cultivation objective. To maximize biomass accumulation and energy efficiency, the constant regime is most suitable. However, if the focus is on nutritional quality and the production of bioactive compounds, such as carotenoids, the Gaussian regime offers advantages.

Therefore, it is observed that the strategic combination of lighting regimes and light spectra can optimize both the yield and quality of harvests.

## Data Availability

The raw data supporting the conclusions of this article will be made available by the authors, without undue reservation.
