# Supplementary material for: Growing in red: impact of different light spectra and lighting conditions on lentil microgreens growth in vertical farming
Source: Front Plant Sci. 2024 Dec 23;15:1515457. doi: 10.3389/fpls.2024.1515457 (PMC11700821; doi:10.3389/fpls.2024.1515457)
Supplement: Supplementary file 1 [file DataSheet1.pdf]

Supplementary Material

SUPPLEMENTAL DATA

1 SUPPLEMENTARY TABLES AND FIGURES

Color	Blue	Std. Error	White	Std. Error	RBW	Std. Error	Red	Std. Error	CV
**NPQ**	0.448 a	±0.0063	0.413 b	±0.0085	0.4045 bc	±0.0136	0.4022 c	±0.0124	5.88%
**Y(II)**	0.559 c	±0.0064	0.5907 b	±0.0048	0.6335 a	±0.0104	0.6395 a	±0.0102	3.51%
**HMD**	0.0529 b	±0.0016	0.0543 c	±0.0046	0.0636 b	±0.0052	0.0849 a	±0.0022	11.6%
**Fv/Fm**	0.7946 a	±0.0020	0.7947 a	±0.0017	0.8056 a	±0.0280	0.7933 a	±0.0283	4.51%
**ETR**	3.3545 a	±0.0307	3.1625 b	±0.0250	3.4667 a	±0.0494	3.3583 a	±0.0615	3.65%

Table S1. Mean values and standard errors for NPQ, Y(II), HMD, and ETR according to light spectrum (Color). No interaction effect was observed for these variables, with significant main effects of light regime and light spectrum. Higher values were observed for NPQ and HMD under blue and red light, respectively, and lower values for NPQ and Y(II) under red and RBW light. For ETR, white light showed lower values compared to the other spectra, which did not differ significantly from each other.

Regime	Constant	Std. Error	Gaussian	Std. Error	CV
**NPQ**	0.421 a	±0.014	0.395 b	±0.016	5.88%
**Y(II)**	0.583 b	±0.012	0.610 a	±0.011	3.51%
**HMD**	0.071 a	±0.005	0.052 b	±0.005	11.6%
**Fv/Fm**	0.80 a	±0.028	0.78 a	±0.028	4.51%
**ETR**	3.136 b	±0.062	3.354 a	±0.061	3.65%

Table S2. Mean values and standard errors for NPQ, Y(II), HMD, and ETR according to light regime (Constant and Gaussian). No interaction effect was observed for these variables, with significant main effects of light regime. Higher values were observed for NPQ and HMD under the constant light regime, while lower values were observed for Y(II) and ETR under the constant light regime when compared to the Gaussian regime.

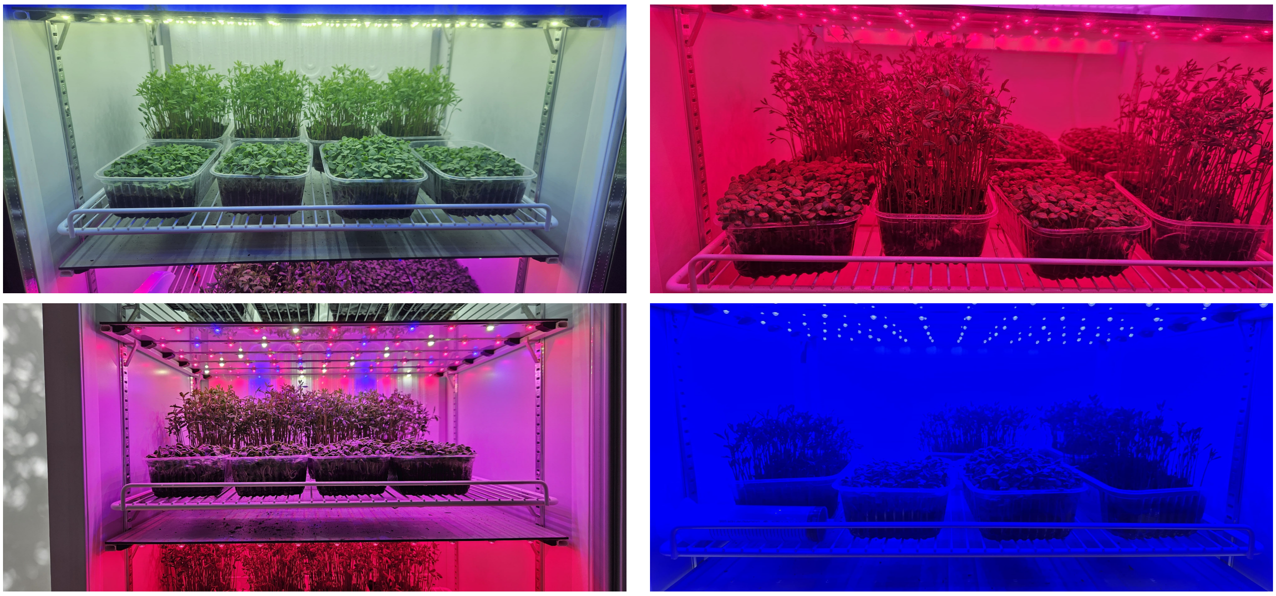

Figure S1. Plants subjected to white, red, RBW, and blue light treatments in the growth chamber (Spectral Int®, Rio Verde, Brazil).

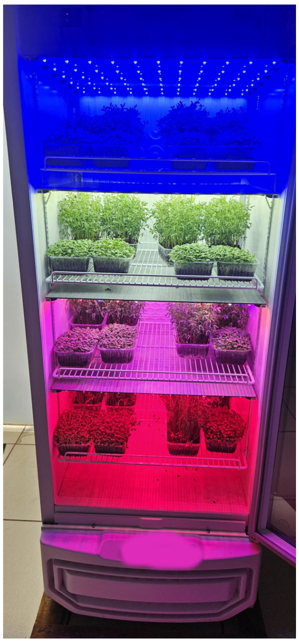

Figure S2. Growth chamber from Spectral Int® (Rio Verde, Brazil), used for microgreen production at the Goiano Federal Institute of Education, Science and Technology, Rio Verde, Brazil.

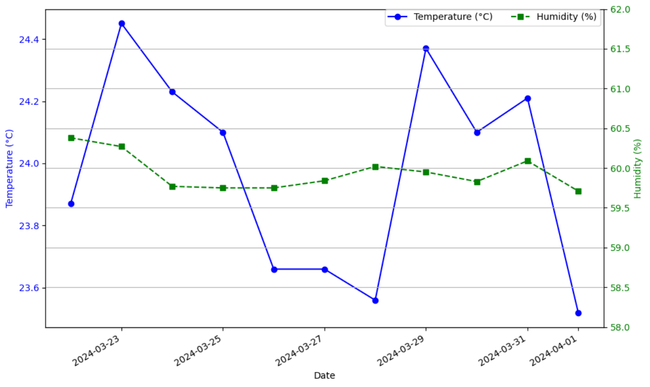

Figure S3. Graph showing temperature and humidity variations over time under Gaussian curve light treatment. The x-axis represents time, while the y-axis shows the corresponding temperature (°C) and relative humidity (%RH)

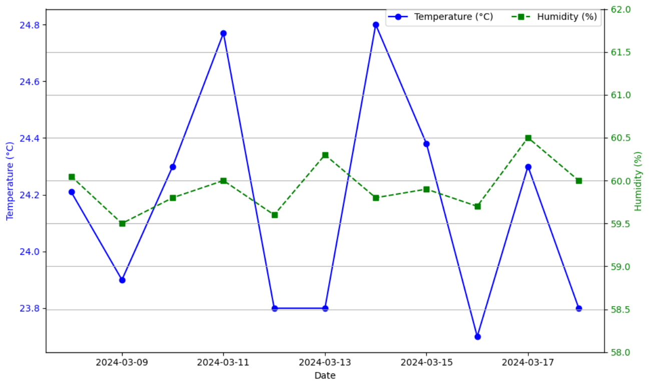

Figure S4. Graph showing temperature and humidity variations over time under constant light treatment. The x-axis represents time, while the y-axis shows the corresponding temperature (°C) and relative humidity (%RH)

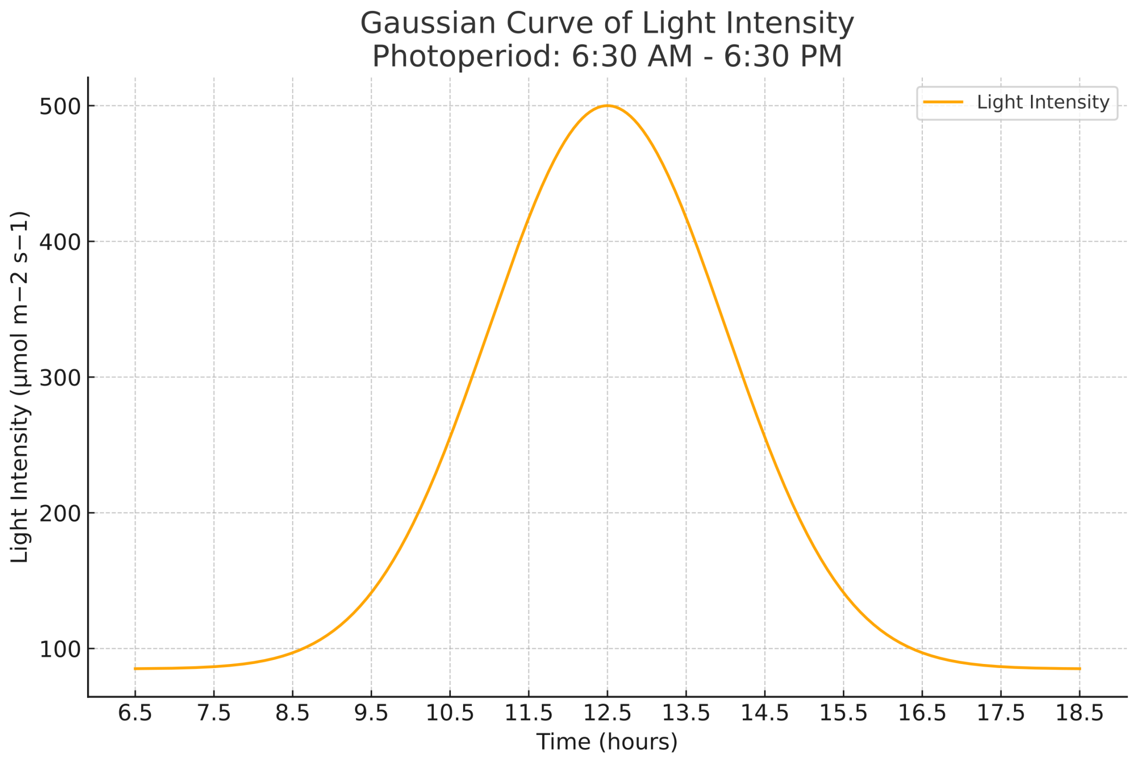

Figure S5. Gaussian curve representing the variation of light intensity during a 12-hour photoperiod (6:30 AM to 6:30 PM). The curve illustrates the gradual increase in light intensity from 85 to 500 µmol m^{-2} s^{-1}, peaking at midday and decreasing towards the end of the photoperiod.
